# Relative Importance of Different Elements of Mitochondrial Oxidative Phosphorylation in Maintaining the Barrier Integrity of Retinal Endothelial Cells: Implications for Vascular-Associated Retinal Diseases

**DOI:** 10.3390/cells11244128

**Published:** 2022-12-19

**Authors:** Shaimaa Eltanani, Thangal Yumnamcha, Andrew Gregory, Mahmoud Elshal, Mohamed Shawky, Ahmed S. Ibrahim

**Affiliations:** 1 Department of Ophthalmology, Visual, and Anatomical Sciences, Wayne State University, Detroit, MI 48201, USA; 2 Department of Pharmacology and Toxicology, Faculty of Pharmacy, Mansoura University, Mansoura 35516, Egypt; 3 Department of Pharmacology, Wayne State University, Detroit, MI 48201, USA; 4 Department of Biochemistry, Faculty of Pharmacy, Horus University, New Damietta 34518, Egypt; 5 Department of Biochemistry, Faculty of Pharmacy, Mansoura University, Mansoura 35516, Egypt

**Keywords:** human retinal endothelial cells (HRECs), rotenone, oligomycin, FCCP, oxidative phosphorylation, OxPhos, capacitance, impedance, ECIS modeling, Rb resistance, α resistance, barrier integrity

## Abstract

Purpose: Mitochondrial dysfunction is central to breaking the barrier integrity of retinal endothelial cells (RECs) in various blinding eye diseases such as diabetic retinopathy and retinopathy of prematurity. Therefore, we aimed to investigate the role of different mitochondrial constituents, specifically those of oxidative phosphorylation (OxPhos), in maintaining the barrier function of RECs. Methods: Electric cell-substrate impedance sensing (ECIS) technology was used to assess in real time the role of different mitochondrial components in the total impedance (Z) of human RECs (HRECs) and its components: capacitance (C) and the total resistance (R). HRECs were treated with specific mitochondrial inhibitors that target different steps in OxPhos: rotenone for complex I, oligomycin for complex V (ATP synthase), and FCCP for uncoupling OxPhos. Furthermore, data were modeled to investigate the effects of these inhibitors on the three parameters that govern the total resistance of cells: Cell–cell interactions (R_b_), cell–matrix interactions (α), and cell membrane permeability (Cm). Results: Rotenone (1 µM) produced the greatest reduction in Z, followed by FCCP (1 µM), whereas no reduction in Z was observed after oligomycin (1 µM) treatment. We then further deconvoluted the effects of these inhibitors on the R_b_, α, and C_m_ parameters. Rotenone (1 µM) completely abolished the resistance contribution of R_b_, as the R_b_ became zero immediately after the treatment. Secondly, FCCP (1 µM) eliminated the resistance contribution of R_b_ only after 2.5 h and increased C_m_ without a significant effect on α. Lastly, of all the inhibitors used, oligomycin had the lowest impact on R_b_, as evidenced by the fact that this value became similar to that of the control group at the end of the experiment without noticeable effects on C_m_ or α. Conclusion: Our study demonstrates the differential roles of complex I, complex V, and OxPhos coupling in maintaining the barrier functionality of HRECs. We specifically showed that complex I is the most important component in regulating HREC barrier integrity. These observed differences are significant since they could serve as the basis for future pharmacological and gene expression studies aiming to improve the activity of complex I and thereby provide avenues for therapeutic modalities in endothelial-associated retinal diseases.

## 1. Introduction

Breakdown of the inner endothelial blood–retinal barrier (iBRB) is a primary cause of retinal edema and consequent vision loss in vascular eye diseases. Retinal endothelial cells (RECs) are the principal component of iBRB, and their cytoplasm is filled with numerous mitochondria and endoplasmic reticulum (ER) [1]. Unique characteristics of RECs compared to other endothelial cell types are the lack of fenestrations and the existence of stable tight junctions between cells imposed by the zonula occludens [1]. These features strengthen the iBRB, which eliminates circulating solutes under physiological conditions from entering the retina [2]. To this end, numerous studies demonstrated the evidence that endothelial dysfunction plays a vital role in the pathogenesis of several ischemic retinal diseases, including diabetic retinopathy, retinopathy of prematurity, and occlusion of the central retinal artery [3]. A significant amount of work has been undertaken to define operative mechanisms, and recently the importance of mitochondria in maintaining endothelial functions has been given considerable attention, although the dependency of endothelium on glycolysis for ATP production [4].

Mitochondria are central bioenergetic hotspots generating the bulk of ATP needed for cellular functions through the oxidative phosphorylation (OxPhos) process. OxPhos is a coupled event between electron transport and ATP synthesis that takes place at mitochondrial cristae. Cristae are folds in the inner mitochondrial membrane (IMM) harboring four electron transport complexes (complexes I to IV) and ATP synthase (complex V). The electron transport chain (ETC) is initiated by a sequential passage of electrons from the Krebs cycle electron donors (NADH and FADH2) to complexes I and II, respectively, to complex III, then to complex IV, and ultimately to the molecular oxygen (O_2_). As electrons move through the ETC, hydrogen ions are also pumped from the mitochondrial matrix into the mitochondrial intermembrane space (IMS) by complexes I, III, and IV, producing an electrochemical gradient. The Nobel laureate Peter Mitchell [5] proposed a chemiosmotic theory whereby the generated electrochemical gradient constitutes the driving force for ATP synthesis by re-entry of hydrogen ions into the mitochondrial matrix through complex V, which has two major functional subunits designated as *F_0_* and *F_1_*. The *F_0_* subunit is embedded in the IMM and contains a proton-powered motor protein, whereas the *F_1_* subunit is localized inside the mitochondrial matrix and contains a catalytic site for synthesizing ATP from Pi and ADP. Two stalks structurally link the *F_0_* and *F_1_* subunits; the *F_0_* stalk, which is static peripheral, and the *F_1_* stalk, which is central and rotatory, and conveys the swirl from the F_0_ subunit to the *F_1_* subunit [6]. The antibiotic oligomycin has been proven to be a valuable tool in studying the contribution of complex V to various cellular processes as it binds the upper part of the F_0_ stalk containing an oligomycin sensitivity conferral protein (OSCP), thereby inhibiting complex V activity [7]. However, the relative importance of complex V in the maintenance of retinal endothelial barrier integrity has never been examined.

In addition to complex V, the largest protein families that exist in mitochondria include complex I (NADH: ubiquinone reductase) of the ETC, consisting of 45 subunits [7,8]. X-ray crystallography studies [8] revealed the three-dimensional structure of complex I as an L-shaped molecule with one arm embedded in the IMM housing 3–4 proton-pumping modules, while the other arm extends inside the mitochondrial matrix to facilitate the passage of electrons from NADH [9]. Rotenone is a classical inhibitor of complex I that has been used to understand the role of complex I in different cellular functions. Rotenone blocks electron flow through complex I to the downstream coenzyme Q, causing electron accumulation inside the mitochondrial matrix and subsequent formation of reactive oxygen species (ROS) [10,11], which play physiological and pathological roles in maintaining cellular structure and functions. Although the loss of complex I activity can be compensated by increased complex II activity in most cells [12], *the importance of complex I in maintaining REC barrier assembly has not been* addressed.

In general, proton translocation across the IMM during ETC is crucial in coupling ETC to ATP production. However, uncoupling the two processes may be biologically beneficial in specific situations. For example, increased expression of uncoupling proteins (UCP) in brown adipose tissue causes protons to leak across the IMM, thus collapsing the chemiosmotic gradient, dissipating heat from the metabolic energy, and resulting in thermogenesis rather than ATP generation. Carbonyl cyanide-p-triflouromethoxyphenyl hydrazone (FCCP) is another well-known uncoupler that is used pharmacologically to determine the role of uncoupling OxPhos from ATP synthesis in cell functionality [12,13]. In relation to REC, the contribution of uncoupling OxPhos from ATP production to the barrier integrity is largely unknown.

When a cell is confronted with dysregulated mitochondrial activity and energy alteration, it responds in various ways that are ultimately reflected in dynamic changes in cell behavior. The electric cell-substrate impedance sensing (ECIS) system is well positioned to examine how the cells dynamically respond to mitochondrial insults because it monitors the cell monolayer’s total electrical impedance in real time. Additionally, the ECIS technology has the ability to dissect multiple components of the REC barrier at both the paracellular and basolateral surfaces as well as at membrane capacitance and subsequent cell morphology [14,15]. Previously, we have shown that the components of mitochondrial OxPhos maintain the functionality of retinal pigment epithelium, a principal constituent of the outer BRB, in a differential manner. Therefore, we hypothesized that OxPhos components contribute differentially to maintaining the functionality of RECs, the main component of the iBRB. In the current study, we pursued to analyze in more detail the relative importance of the aforementioned mitochondrial components in regulating the behavior of human RECs, including integrity of the barrier, quality of intercellular junctions, quality of extracellular matrix adhesions, and the morphology of cell membrane in real time using the ECIS technology.

## 2. Materials and Methods

### 2.1. Human Retinal Endothelial Cells (HRECs)

HRECs isolated from healthy donors were purchased from Cell Systems (Kirkland, WA, USA) and cultured in full media of Microvascular Endothelial Cell Growth Medium-2 (EBM2; Lonza, Walkersville, MD, USA (Cat. #: CC-3202 EGM-2MV)) supplemented with growth factors in a humidified incubator with 5% CO_2_ at 37 °C as described previously [16]. After reaching confluence, the culture media of HRECs were replaced with media freed from serum and various growth factors overnight. After treatment with different concentrations of mitochondrial inhibitors, the HRECs were monitored by the ECIS technology for 30 h.

### 2.2. ECIS Modeling for Measurement of HREC Barrier Function

The effect of different inhibitors targeting different steps in mitochondrial OxPhos on the cellular barrier functionality of HRECs was evaluated by measuring the overall impedance [Z; ohms (Ω)] using ECIS^®^Zθ (theta) biosensor technology (Applied Biophysics Inc., Troy, NY, USA) followed by mathematical modeling as described previously [16,17]. Before seeding the cells, the electrode substrate array of the 96-well plate (96W20idf PET, Applied Biophysics, Inc.) was prepared by coating with 50 µL of 100 µM cysteine (Applied Biophysics) for 30 min, followed by aspiration. The cysteine-coated electrode array was additionally coated with 50 µL of 0.02% gelatin (Sigma; G1393, Burlington, MA, USA) for another 30 min, followed by aspiration. Then, HRECs were cultured in full media of EBM2 at a cell density of 30,000 cells per well until the cells reached confluence. After the HRECs achieved a confluent monolayer, which was indicated by reaching a steady-state capacitance below 20 nF, the culture media were replaced by media free from serum and various growth factors for overnight. Then, the HRECs were treated with the following mitochondrial inhibitors: FCCP (Agilent, Santa Clara, CA, USA, 1 and 10 µM), which uncouples proton gradient across ETC; oligomycin (Sigma, 1 and 10 µM), which inhibits ATP synthase (complex V); and rotenone (Sigma, 1 and 10 µM), which disrupts ATP synthesis by inhibiting complex I. Control HRECs were treated with vehicle (DMSO, Sigma). The Z across the HRECs monolayer was measured as a function of time and frequency after applying an alternating current (AC) of 1 µA to the electrode array embedded in the bottom of the 96-well plate. Nine frequencies of AC with a range from 250 to 64,000 Hz were used in measuring the Z. The normalized Z value of each time point was obtained by dividing the raw Z value at each time point by the Z value obtained before treatment with mitochondrial inhibitors. Then, the normalized Z values were plotted with respect to time. Furthermore, ECIS software was used to calculate the resistance and capacitance across HREC monolayer from the measured Z. Finally, ECIS software was used to mathematically model the total resistance across the HREC monolayer into three parameters: R_b_, which signifies the strength of the paracellular resistance between HRECs (ohms-cm^2^); α, which signifies the degree of basolateral resistance of HRECs to the extracellular matrix (ohms-cm^1/2^), and C_m_ signifies the capacitance at the HRECs membrane (µF/cm^2^). To study the differential response of HRECs after treatment with various mitochondrial inhibitors throughout the whole experimental period, the area under the curve (AUC) of the normalized parameter was calculated.

### 2.3. Assessment of Mitochondrial Inhibitors on HREC Viability

The viability of HRECs was measured by the lactate dehydrogenase (LDH) Cytotoxicity assay (CyQUANT™; Invitrogen-C20300, Waltham, MA, USA). Briefly, HRECs were cultured in a 96-well plate (3 × 10^4^/200 μL/well) as described above and then treated with FCCP (1 and 10 μM), oligomycin (1 and 10 μM), rotenone (1 and 10 μM), vehicle (DMSO) for 24 and 48 h. After treatment, the supernatants were collected, mixed with an LDH reaction mixture, and incubated at room temperature for 30 min. The absorbance was then measured spectrophotometrically using a microplate reader (Synergy HI Hybrid Reader, BioTek, Winooski, VT, USA). The amount of LDH released in the media was calculated by subtracting the background absorbance of 680 nm from the 490 nm absorbance per the manufacturer’s instructions.

## 3. Statistical Analysis

Analytical comparison of the experimental groups was assessed using the two-tailed *t*-test or one-way analysis of variance (ANOVA). For the correction of multiple comparisons, the Bonferroni correction was used. Graphical representations of *p* values are * *p* ≤ 0.05, ** *p* ≤ 0.01, *** *p* ≤ 0.001, and **** *p* ≤ 0.0001. The figure legends include the number of biological replicates.

## 4. Results

### 4.1. Real-Time Monitoring of the Effect of Different Mitochondrial Components on HREC Barrier Function

**Figure 1:**
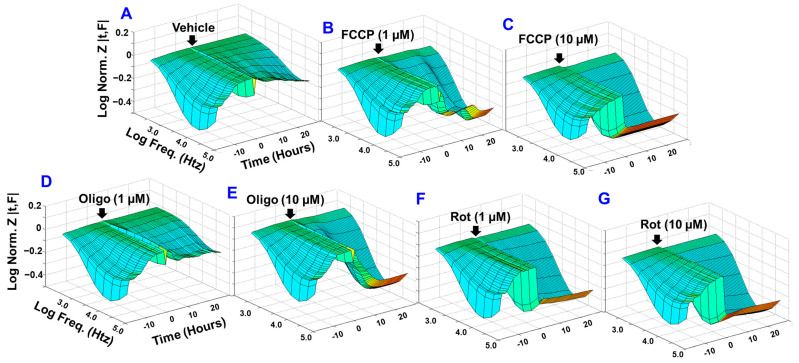
Bioimpedance analysis of the effects of different mitochondrial inhibitors on the barrier function of HRECs using the ECIS technology. Three-dimensional analysis was prepared by plotting the log of normalized impedance (*Y*-axis) as a function of both log frequencies of 1 µA alternating current (AC) on the *X*-axis and time on the *Z*-axis. HRECs were treated with the vehicle (DMSO; **A**) and different concentrations of mitochondrial inhibitors: FCCP (1 and 10 µM; **B** and **C**, respectively), oligomycin (Oligo;1 and 10 µM; **D** and **E**, respectively), and rotenone (Rot, 1 and 10 µM; **F** and **G**, respectively) at the time point when HRECs formed a confluent monolayer. This time was considered to be (t = 0), and its impedance value (Z_0_) was used to normalize all other impedance measurements (Z_t_) by determining the ratio of Z_t_/Z_0_. Different AC frequencies ranging from 250 to 64,000 Hz were utilized for the real-time impedance measurement. Abbreviations: Z, impedance; Norm, normalized; Freq, frequency; Z_t_, the impedance at time t; Z_0_, the impedance at time 0. Data shown are representative three-dimensional plots of six independent biological replicates per group.

Considering that impaired barrier function of RECs contributes to the pathogenesis of various ischemic retinal diseases, bioimpedance analysis was performed *in vitro* to determine whether different mitochondrial components have varying importance in maintaining REC barrier function. The selected inhibitors were carbonyl cyanide-p-trifluoromethoxyphenyl hydrazon (FCCP; Figure 1B,C) for uncoupling ETC from the associated ATP synthesis, oligomycin (Figure 1D,E) for inhibiting ATP synthase (complex V), and rotenone for inhibiting complex I (Figure 1F,G). Various concentrations of these mitochondrial inhibitors were added to HRECs when cells reached a plateau in the impedance (Z) curve (the *y*-axis in the 3D model), an indicator of a confluent monolayer. After that, the barrier integrity was continuously monitored by the ECIS technology over a 30 h period (the *z*-axis in the 3D model) and throughout a frequency range between 250 and 64,000 Hz (the *x*-axis in the 3D model). Based on visual representations of the Z as a function of time and AC frequency (Figure 1A–G), higher concentrations of these inhibitors (10 µM) caused dramatic reductions in the corresponding Z curves and thus the barrier integrity of HRECs when compared to the vehicle-treated control group. However, at a lower concentration (1 µM) of these inhibitors, rotenone displayed the greatest reduction in Z followed by FCCP, whereas no clear reduction in Z was observed after oligomycin treatment when compared to the control group. These results demonstrate differential roles for complex I, complex V, and coupling of OxPhos in maintaining the barrier functionality of HRECs, which have been investigated in more depth by the following experiments.

### 4.2. The Effect of OxPhos Uncoupling on the Behavior of HRECs

**Figure 2:**
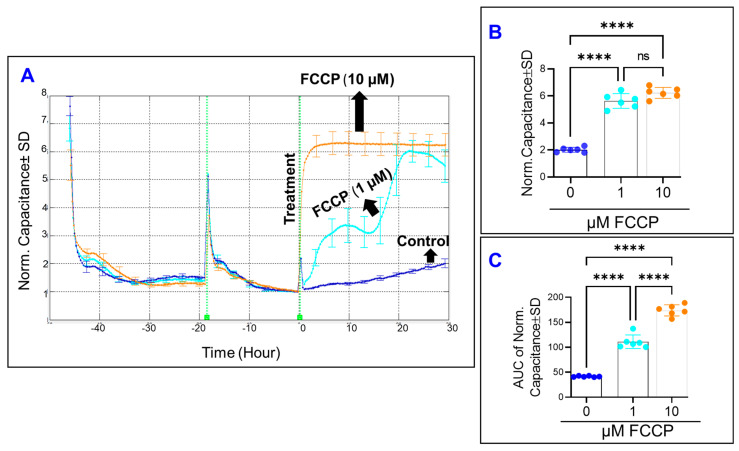
Real-time measurement of human retinal endothelial cells’ spreading capacity treated with OxPhos uncoupler (FCCP). (**A**) The spreading of HRECs over the electrode under the treatment of DMSO (vehicle) or FCCP (1 and 10 µM) was measured at an AC frequency of 64,000 Hz and represented as normalized capacitance vs. time. The capacitance value at time 0 (C_0_) was used to normalize all other capacitance measurements. (**B**) The bar graph represents each group’s normalized capacitance at the time (t = 30 h). (**C**) The bar graph represents each group’s area under the normalized capacitance curve between the time interval t = 0–30 h. Statistical analysis was performed using the ANOVA test followed by the Tukey post hoc test. Abbreviations: Norm, normalized; AUC, area under the curve. ****, *p* ≤ 0.0001; ns, no significance.

Mathematically, the total impedance of HRECs represents two components—the capacitance (C) and the resistance (R)—which are used as indicators for different cell behaviors. The C reflects the cells’ spreading over the substrate, while the R describes the strength of the cell–cell junction and cell–matrix adherence as well as changes in cell morphology. Therefore, we sought to determine the importance of maintaining the coupling of OxPhos on these cells’ parameters using the uncoupling agent FCCP. First, we determined the effect of FCCP on the spreading movement of HRECs over the substrate by monitoring the C across the cells (Figure 2A). To accomplish this, the optimum frequency that corresponds to the maximum HRECs’ spreading over the electrode was chosen to be 64,000 Hz based on our previous publication [16]. Since the C across the cells decreases in inverse proportion to the spreading extent, determining the effects of FCCP on HRECs’ spreading was best carried out once the cells become electrically quiescent, which was defined as the reaching of the stable minimum plateaus in C curves after placement of HRECs on the electrode (Figure 2A). After FCCP (10 µM) was added to HRECs, we observed that C rapidly rose to a value that was 6-fold higher than that of the control group, and then plateaued for the remainder of the experiment (Figure 2A). On the other hand, it took 20 h of FCCP (1 µM)-treated HRECs to reach the same C of HRECs treated with FCCP (10 µM). Furthermore, HRECs treated with 1 µM of FCCP showed a characteristic behavior of increasing C from baseline then reaching a momentary maximum followed by another increase for the remainder of the experiment. This likely represents dynamic changes in the spreading behavior of HRECs over the electrode in response to the treatment with FCCP (1 µM). By the end of the experiment, both FCCP concentrations increased the C of HRECs to the same extent without showing an obvious dose–response effect (Figure 2B). However, by calculating the area under the curve (AUC) for each of the C curves, there was a significant difference in AUC values for each FCCP treatment throughout the entire experiment, indicating a dose-dependent effect of OxPhos uncoupling on C and on the spreading of HRECs over the substrate (Figure 2C).

**Figure 3:**
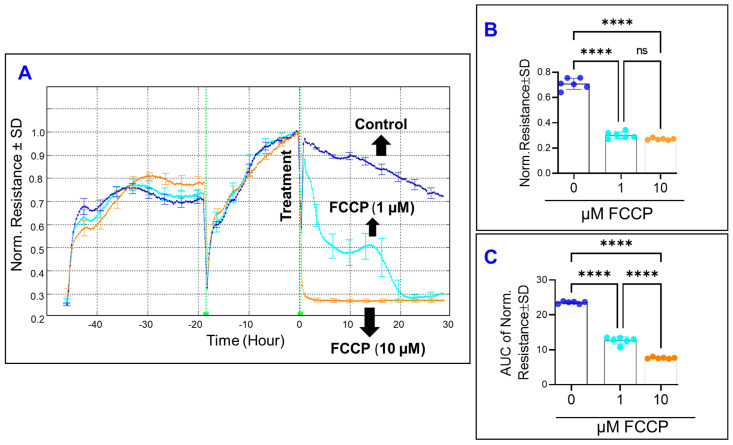
Real-time measurement of human retinal endothelial cells’ total resistance treated with OxPhos uncoupler (FFCP). (**A**) The total resistance across HRECs after treatment with vehicle (DMSO) or FCCP (1 and 10 µM) was represented by plotting normalized resistance vs. time, measured at an AC frequency of 4000 Hz. When treatment started, the resistance value at time 0 (R_0_) was used to normalize all other resistance measurements. (**B**) The bar graph represents each group’s normalized resistance at the endpoint (t = 30 h). (**C**) The bar graph represents each group’s area under the normalized resistance curve between the time interval t = 0–30 h. Statistical analysis was performed using the ANOVA test followed by the Tukey post hoc test. Abbreviations: Norm, normalized; AUC, area under the curve; ****, *p* ≤ 0.0001; ns, no significance.

We subsequently assessed the effect of OxPhos uncoupling on the total electrical R across the HRECs as an index of their overall barrier functionality. To achieve this goal, the optimum frequency corresponding to the maximum total R was chosen to be 4000 Hz based on our previous study [16]. HRECs were then treated with FCCP and the total R was measured for 30 h after treatment. As shown in Figure 3A, and in contrast to the effect of FCCP on the C, FCCP at 1 µM exhibited a characteristic R curve which started with a decrease in the initial R reading until reaching a momentary minimum, then decreased again to a minimum R, and then plateaued for the remainder of the experiment. However, treatment with a higher concentration of FCCP (10 µM) caused an immediate and sharp decrease in the R curve, followed by reaching a plateau phase as early as 1–2 h. Both concentrations of FCCP caused statistically significant decreases in HRECs’ R at the end of the experiment, but without a clear dose-related trend (Figure 3B). Instead, a dose-dependent effect of FCCP on decreasing HRECs’ R was seen by calculating the AUC (Figure 3C), where each treatment group experienced decreases in R at a significantly different rate.

**Figure 4:**
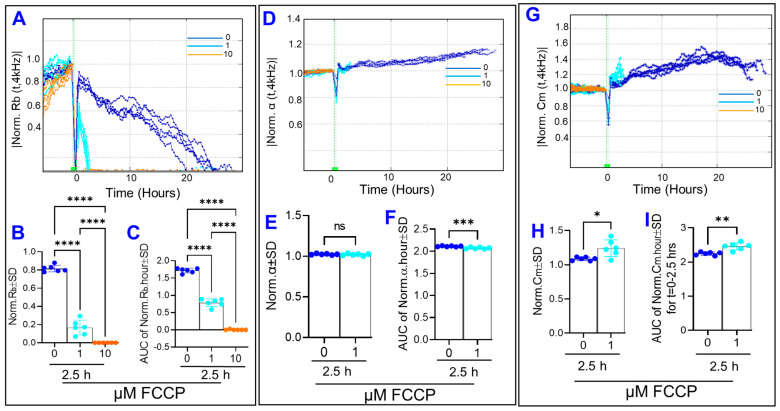
Real-time monitoring of human retinal endothelial cells’ α, R_b_, and C_m_ treated with OxPhos uncoupler (FCCP). (**A**) Normalized R_b_ curve measured at 4000 Hz vs. time (t). (**B**) The bar graph represents normalized R_b_ measured at t = 2.5 h. (**C**) AUC of normalized R_b_ measured between 0 and 2.5 h. (**D**) Normalized α curve measured at 4000 Hz vs. time (t). (**E**) The bar graph represents normalized α measured at t = 2.5 h. (**F**) AUC of normalized α measured between 0 and 2.5 h. (**G**) Normalized C_m_ curve measured at 4000 Hz vs. time (t). (**H**) The bar graph represents normalized C_m_ measured at t = 2.5 h. (**I**) AUC of normalized C_m_ measured between 0 and 2.5 h. Statistical analysis was performed using the ANOVA test followed by the Tukey post hoc test. Abbreviations: Norm, normalized; AUC, area under the curve; ns, no significance; *, *p* ≤ 0.05; **, *p* ≤ 0.01; ***, *p* ≤ 0.001; ****, *p* ≤ 0.0001; n = 5–6/group.

After having shown that uncoupling OxPhos alters the barrier functionality of HRECs, interest has been expanded to dissect the effect of the OxPhos uncoupling on the three parameters that govern the total resistance of cells, which include: R_b_ (the intercellular resistance); α (the basolateral adhesion of HRECs to the substrate) and C_m_ (the capacitance at the cell membrane). To this end, the Giaever and Keese differential mathematical model [18] was applied, and the effects of FCCP on these three components were depicted in Figure 4. Firstly, Figure 4A shows the time-dependent effect of OxPhos uncoupling on normalized R_b_. FCCP at 10 µM eliminated the resistance contribution of R_b_, as R_b_ for this group reached zero immediately after the treatment and remained constant thereafter. Furthermore, the R_b_ curve of FCCP (1 µM) displayed an interesting pattern of approaching and reaching zero immediately after the treatment, increasing dramatically, and then gradually returning to zero by 2.5 h. In this 2.5 h time interval, FCCP reduced both endpoint R_b_ values (Figure 4B) and behavior of R_b_ throughout this experimental period, represented by the AUC in Figure 4C. Secondly, Figure 4D–F demonstrate changes in the basolateral resistance of HRECs over time after treatment with FCCP using α curves. Since the real values for α cannot be modeled by the ECIS software when the corresponding R_b_ values are zero [18], the α curve for FCCP (10 µM) ended immediately after FCCP addition, and the α curve for FCCP (1 µM) terminated abruptly at 2.5 h, which are the time points when the R_b_ curve for each group reaches 0, as shown in Figure 4A. Accordingly, we were only able to calculate the effect of the lower concentration of FCCP (1 µM) on changes in α within the first 2.5 h post-FCCP treatment. As shown in Figure 4E,F, FCCP at 1 µM caused a small (~5–10%) yet significant decrease in α throughout the experimental period of 2.5 h but not at the end of this experimental period, indicating that the effect of OxPhos uncoupling on reducing the adhesion of HRECs to the substrate is a reversible process. Next, Figure 4G displays C_m_ over time after treatment with FCCP. We similarly observed that the C_m_ curve for FCCP (10 µM) ended immediately after FCCP addition, and the C_m_ curve for FCCP (1 µM) ended abruptly after 2.5 h since C_m_ cannot be modeled by the ECIS software if the corresponding R_b_ is 0. The C_m_ in the FCCP 1 µM group is statistically significantly increased both throughout and at the end of the 2.5 h, whereas C_m_ values of the FCCP 10 µM group could not be calculated during this time interval (Figure 4H,I), indicating that the effect of OxPhos uncoupling on increasing the C_m_ of HRECs is an irreversible process. Taken together, the results shown in Figure 2, Figure 3 and Figure 4 indicate that maintaining OxPhos coupling is necessary for cell spreading and the barrier integrity of HRECs, specifically to strengthen the paracellular junction and to control the capacitance at the cell membrane.

### 4.3. The Effect of ATP Synthase Inhibition on the Behavior of HRECs

**Figure 5:**
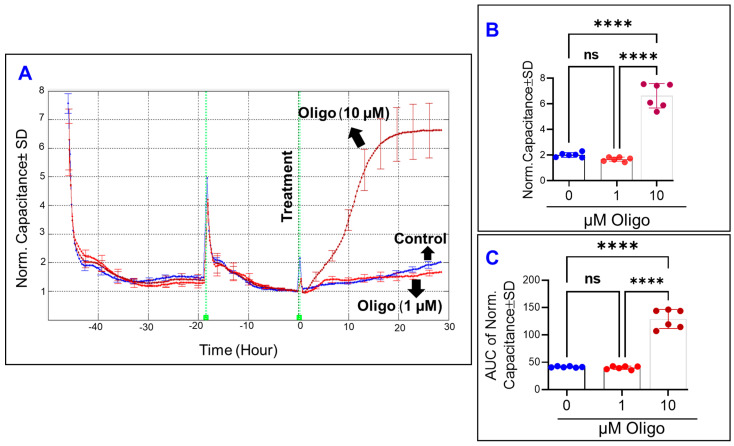
Real-time measurement of human retinal endothelial cells’ spreading capacity treated with an ATP synthase inhibitor (Oligomycin; Oligo). (**A**) The spreading of HRECs across the electrode after treatment with vehicle (DMSO) or Oligo (1 and 10 µM) was represented by plotting normalized capacitance vs. time, measured at an AC frequency of 64,000 Hz. The capacitance value at time 0 (C_0_) was used to normalize all other capacitance measurements. (**B**) The bar graph represents normalized capacitance measured at t = 30 h. (**C**) AUC of normalized capacitance measure at the time interval between 0 and 30 h. Data were analyzed by the ANOVA test followed by the Tukey post hoc test. Abbreviations: Norm, normalized; AUC, area under the curve; ****, *p* ≤ 0.0001; ns, no significance.

**Figure 6:**
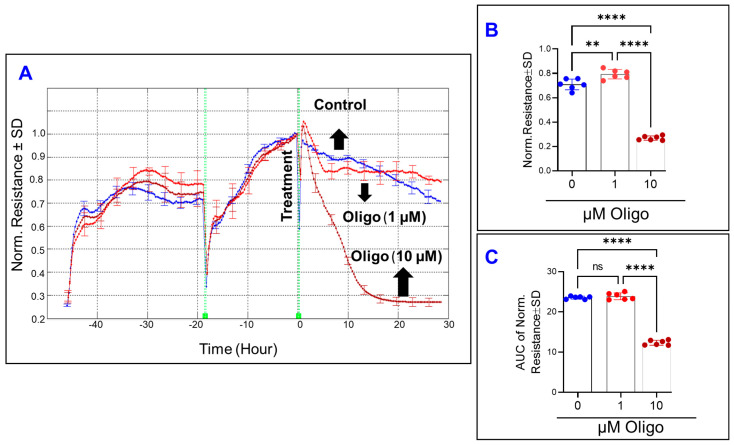
Real-time measurement of human retinal endothelial cells’ total resistance treated with an ATP synthase inhibitor (Oligomycin; Oligo). (**A**) The total resistance was measured across HRECs and represented by plotting normalized resistance vs. time at an AC frequency of 4000 Hz. The resistance value at time 0 (R_0_) was used to normalize all other resistance measurements. (**B**) The bar graph represents normalized resistance measured at the time (t = 30). (**C**) AUC of normalized resistance measured at the time interval between 0 and 30 h. Data were analyzed by the ANOVA test followed by the Tukey post hoc test. Abbreviations: Norm, normalized; AUC, area under the curve; **, *p* ≤ 0.01; ****, *p* ≤ 0.0001; ns, no significance.

**Figure 11:**
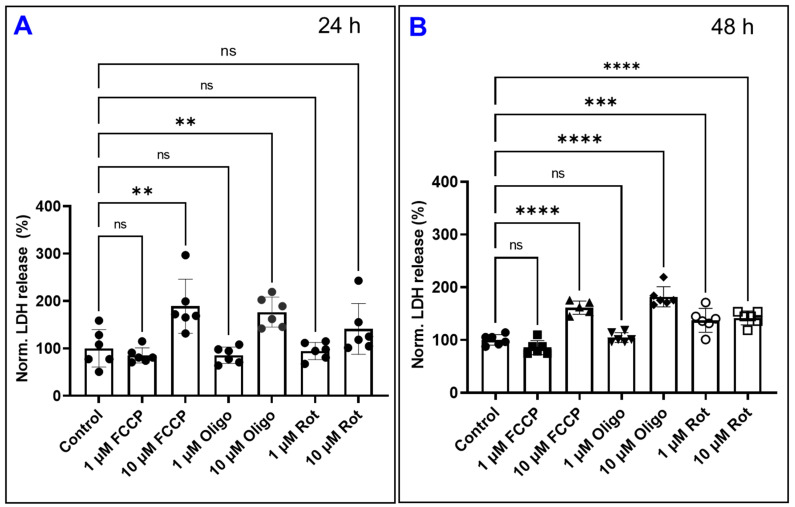
Effects of different mitochondrial inhibitors on HREC viability. The amount of lactate dehydrogenase (LDH) released after treatment with various mitochondrial inhibitors at (**A**) 24 h and (**B**) 48 h. Statistical analysis was performed using the ANOVA test followed by the Tukey post hoc test. Abbreviations: Norm, normalized; ****, *p* ≤ 0.0001; ***, *p* ≤ 0.001; **, *p* ≤ 0.01; ns, no significance.

In contrast to the effects of inhibiting OxPhos coupling by FCCP (1 µM) on increasing the C of HRECs, inhibition of ATP synthase by oligomycin (1 µM) did not affect the capacitance of HRECs and thus the spreading behavior of HRECs neither at the end of the experiment nor throughout the entire experiment (Figure 5A–C). Furthermore, it took ~20 h for the C of HRECs treated with a higher concentration of oligomycin (10 µM) to reach the C of FCCP (10 µM). When the AUCs are compared in Figure 5C, only oligomycin at 10 µM causes a significant increase in the C throughout the entire experiment compared to the control. These data indicate that inhibition of ATP synthase affects the spreading movement of HRECs only at higher concentrations when it becomes toxic (Figure 11). Next, we measured the R parameter of the Z across the monolayer at 4000 Hz, a frequency at which the R is greatest [16], to determine if inhibiting ATP synthase impacts the barrier function of HRECs. As shown in Figure 6A, treating HRECs with oligomycin resulted in a decline in R over time only at a higher concentration (10 µM), where it took 12–15 h for the R to reach a minimum level and then plateaued over time. Although oligomycin (10 µM) reduced both endpoint R values (Figure 6B) and the behavior of R throughout the experiment, the 1 µM oligomycin group did not have a considerable loss in the R compared to the control group throughout the experiment, indicated by the AUC in Figure 6C.

**Figure 7:**
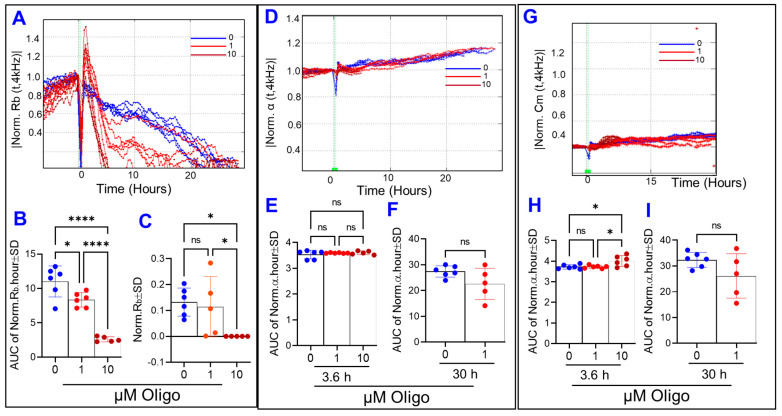
Real-time monitoring of human retinal endothelial cells’ α, R_b_, and C_m_ treated with an ATP synthase inhibitor (Oligomycin; Oligo). (**A**) Normalized R_b_ curve measured at 4000 Hz vs. time (t). (**B**) AUC of normalized R_b_ measured between 0 and 30 h. (**C**) The bar graph represents normalized R_b_ measured at t = 30 h. (**D**) Normalized α curve measured at 4000 Hz vs. time (t). (**E**) and (**F**) AUC of normalized α measured between (0–3.6 h) and (0–30 h), respectively. (**G**) Normalized C_m_ curve measured at 4000 Hz vs. time (t). (**H**,**I**) AUC of normalized C_m_ measured between (0–3.6 h) and (0–30 h), respectively. Data were analyzed by the ANOVA test followed by the Tukey post hoc test. Abbreviations: Norm, normalized; AUC, area under the curve; ns, no significance; * *p*, ≤ 0.05; **** *p*, ≤ 0.0001; n = 5–6/group.

These changes in the R curves were then deconvoluted to test the effect of ATP synthase inhibition on the three main components of barrier integrity: R_b_, α, and C_m_. Firstly, our results showed that R_b_ parameter of the HREC barrier was the parameter that declined first and was most significantly affected by oligomycin (Figure 7A) and in a dose-dependent manner throughout the experimental period (Figure 7B). However, the decrease in R_b_ throughout the experiment in response to oligomycin (1 µM) was normalized at the end of the experiment (Figure 7C), indicating that viable HRECs have the potential to overcome the effect of ATP synthase inhibition on reducing the paracellular junction between the cells. Secondly, α curves in Figure 7D for 1 and 10 µM of oligomycin were terminated at (28–30 h) and (3.2–3.6 h), respectively, which are time periods when the corresponding R_b_ values become zero (Figure 7A). During these experimental periods and before α curves ended, none of the oligomycin concentrations were significantly affecting α values (Figure 7E,F). Thirdly, Figure 7G shows C_m_ over time following treatment with oligomycin, and again only the C_m_ curve for 10 µM of oligomycin was abruptly terminated at 3.2–3.6 h because, as previously mentioned, ECIS cannot evaluate actual values for C_m_ when R_b_ is zero. Considering the AUC before C_m_ curves ended, only the toxic concentration of oligomycin (10 µM) significantly affected C_m_ values, whereas oligomycin at 1 µM had no effect on C_m_ neither at the end of the experiment nor throughout the entire experiment (Figure 7H,I). Collectively, the results in Figure 5, Figure 6 and Figure 7 demonstrate that ATP synthase inhibition impacts the integrity of the paracellular junctions between HRECs since only the R_b_ component responded in a dose-dependent manner. However, this effect of oligomycin on R_b_ is reversible at lower concentrations.

### 4.4. The Effect of Complex I Inhibition on the Behavior of HRECs

**Figure 8:**
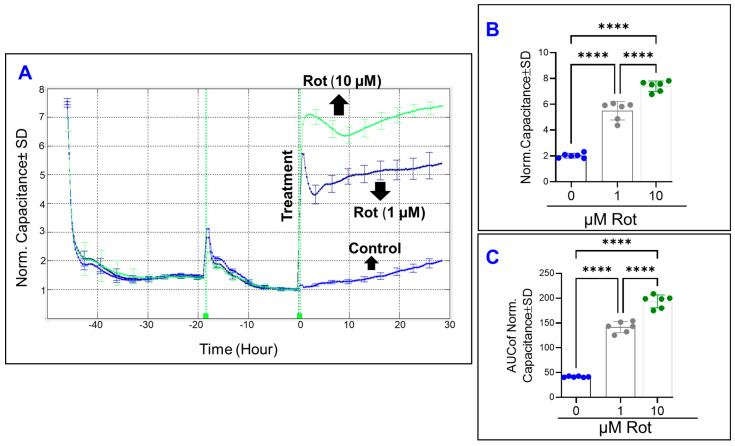
Real-time measurement of human retinal endothelial cells’ spreading capacity treated with an electron transport chain (ETC) complex I inhibitor (rotenone, Rot). (**A**) The spreading capacity of HRECs over the electrode after treatment with vehicle (DMSO) or Rot (1 and 10 µM) was measured at an AC frequency of 64,000 Hz and represented by plotting normalized capacitance vs. time. The capacitance value at time 0 (C_0_) was used to normalize all other capacitance measurements. (**B**) The bar graph represents normalized capacitance measured at the time (t = 30 h). (**C**) AUC of the normalized capacitance measure between the time interval 0 and 30 h. Statistical analysis was performed using the ANOVA test followed by the Tukey post hoc test. Abbreviations: Norm, normalized; AUC, area under the curve; ****, *p* ≤ 0.0001; ns, no significance.

**Figure 9:**
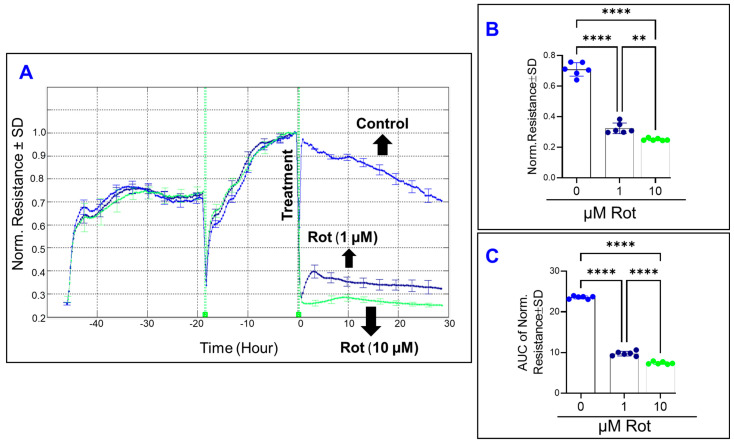
The effect of rotenone on human retinal endothelial cells’ total resistance. (**A**) The total resistance of HRECs after treatment with vehicle (DMSO) or rotenone (Rot; 1 and 10 µM) was measured at an AC frequency of 4000 Hz and represented by plotting normalized resistance vs. time. The resistance value at time 0 (R_0_) was used to normalize all other resistance measurements. (**B**) The bar graph represents each group’s normalized resistance at the time (t = 30 h). (**C**) AUC of the normalized resistance measured at the time interval t = 0–30 h. Statistical analysis was performed using the ANOVA test followed by the Tukey post hoc test. Abbreviations: Norm, normalized; AUC, area under the curve; **, *p* ≤ 0.01; **** *p*, ≤ 0.0001.

**Figure 10:**
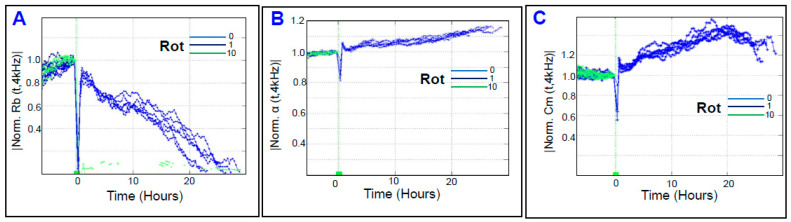
Real-time monitoring of human retinal endothelial cells’ α, Rb, and C_m_ treated with electron transport chain (ETC) complex I inhibitor (rotenone, Rot) measured at 4000 Hz vs. time. (**A**) The curve of normalized R_b_, (**B**) the curve of normalized α, and (**C**) the curve of normalized C_m_.

Next, we monitored the effect of rotenone on the spreading behavior of HRECs by measuring the C response across the cells over time at 64,000 Hz. As shown in Figure 8A, the C of HRECs very rapidly accelerated in a dose-dependent manner after the rotenone treatment. Furthermore, the characteristic C of HRECs treated with rotenone (1 or 10 µM) was observed to increase from baseline, reach a momentary maximum, decelerate for a few hours, and then steadily increase for the rest of the experiment. These changes reflect dynamic changes in the spreading movement of the HRECs in response to the rotenone treatment. At the experimental endpoint of 30 h, rotenone increased C dose dependently (Figure 8B). Moreover, AUC was then calculated for each C curve to determine whether the effects of rotenone on C were dose-dependent over the duration of the experiment. As shown in Figure 8C, the AUCs were still significantly different from each rotenone group, providing evidence that inhibition of complex I in HRECs affects the C dose dependently, and not only at the end but also throughout the entire duration of the experiment. Next, we determined the effect of rotenone on the barrier function of HRECs by monitoring the R response across the HRECs at 4000 Hz over time (Figure 9A). A dose-dependent reduction in the R curve was observed both at the end of the experiment (Figure 9B) and throughout the experimental period (Figure 9C), indicating that inhibition of complex I of the ETC has a significantly damaging and irreversible effect on the REC barrier function. Lastly, we aimed in Figure 10 to elucidate the effect of rotenone on the three components of HRECs’ total resistance; R_b_, α, and C_m_. Figure 10A displays the impact of complex I inhibition on normalized R_b_ over time. Interestingly, both rotenone treatments (1 or 10 µM) completely abolished the resistance contribution of R_b_, as the R_b_ became zero immediately after the treatment and remained constant thereafter. Because R_b_ curves of both rotenone treatments instantly became zeros after the treatment compared to the control group, we were unable to dissect the effect of rotenone on the other resistance components of α and C_m_ (Figure 10B and 10C, respectively). Taken together, the results in Figure 8, Figure 9 and Figure 10 indicate that complex I is an integral component in regulating the barrier functionality and the spreading behavior of HRECs.

### 4.5. The Effect of Different Mitochondrial Inhibitors on the Viability of HRECs

To further ensure that the observed effects of mitochondrial inhibitors on the barrier integrity of HRECs were not a consequence of cell cytotoxicity, we performed a cytotoxicity assay for all HRECs groups by measuring the release of lactate dehydrogenase (LDH). The time points selected were 24 and 48 h post-treatment. At the 24 h mark, only FCCP (10 µM) and oligomycin (10 µM) caused significant increases in LDH release, while FCCP (1 µM), oligomycin (1 µM), and rotenone (1 µM) did not demonstrate any cytotoxicity (Figure 11A). This contrasts with Figure 3A and Figure 9A, where clear reductions to total resistance have already occurred in the FCCP (1 µM) group (Figure 3A) and both rotenone groups (Figure 9A) by this 24 h mark. Especially notable is how the resistances of both rotenone groups have plateaued to a minimum for several hours before this time point, yet Figure 11A shows these groups have not yet lost their viability. However, after 48 h both doses of rotenone have each caused LDH release (Figure 11B). Altogether, these data indicate that disruption of barrier integrity of HRECs is an earlier event that occurs in response to complex I inhibition and OxPhos uncoupling long before any noticeable effects on cell viability.

## 5. Discussion

The main finding of the current study is that the components of mitochondrial OxPhos maintain HREC functionality in a differential manner in which complex I, beyond its role in ATP production, is the most important component in regulating the barrier functionality and the spreading behavior of HRECs. The following results support this conclusion: first, complex I inhibition by rotenone (1 µM) produced the greatest reduction in the electrical impedance of HRECs, followed by uncoupling of OxPhos using FCCP (1 µM), whereas no reduction in the electrical impedance was observed after inhibition of ATP synthase by oligomycin (1 µM). Second, rotenone (1 µM) completely abolished the resistance contribution of R_b_, as the R_b_ became zero immediately after the treatment. In comparison, FCCP (1 µM) eliminated the resistance contribution of R_b_ only after 2.5 h. Third, oligomycin had the lowest impact among these inhibitors on R_b_, which became similar to the control group at the end of the experiment without noticeable effects on C_m_ or α. Lastly, and importantly, the breakdown effect of complex I inhibition on the barrier function of HRECs was not a consequence of cell death. This is evidenced by the fact that at 24 h post-rotenone administration at both 1 and 10 μM concentrations, released LDH levels were not elevated. Additionally, a lack of mitochondrial ATP production likely did not drive the breakdown of barrier integrity when HRECs were treated with rotenone since, as mentioned previously, oligomycin did not decrease electrical impedance and minimally affected R_b_. To our knowledge, this study is the first to demonstrate such temporal relationships between HREC barrier parameters in response to different OxPhos components inhibitors using the ECIS mathematical modeling system.

When considering the three measured components for total impedance, R_b_ was the most profoundly affected in our study since rotenone or FCCP treatment resulted in R_b_ dropping close to or near zero under both conditions. This most likely may be due to direct damage to or reorganization of junctional proteins. However, the decrease in R_b_ with oligomycin (1 µM) treatment was normalized at the end of the experiment (Figure 7C), indicating that HRECs overcome the effect of mitochondrial ATP inhibition on reducing the paracellular junction between the cells. Given the dependency of endothelium on glycolysis for ATP production [4] and the fact that oligomycin induces hyperglycolysis [19], it is very likely that glycolysis compensated the deficit in the ATP-generating capacity of the mitochondria induced by oligomycin. Intriguingly, damage to the mitochondria has been implicated in HREC barrier dysfunction [16], although their ATP-generating capacity could be replaced by glycolysis. Precisely how mitochondria maintain HREC barrier function is still somewhat obscure. Our data in the current study suggest a novel role for mitochondria in maintaining HREC paracellular barrier functionality independent of their ATP-generating capacity, in which complex I followed by OxPhos coupling, are the most important components in regulating the paracellular barrier functionality.

The paracellular barrier has been compromised by a mechanism common to both complex I inhibition and ETC uncoupling, which leads to reactive oxygen species (ROS) accumulating inside the mitochondria, which may result in an oxidative burst and potentially destroying adherent junction proteins (AJPs). It is important to note that not all electrons that pass through ETC, in a normal physiological environment, follow the normal transfer order to O_2_, the final electron acceptor. More specifically, it has been shown that 0.2–2% of the electrons leak out of the ETC and directly interact with oxygen to produce superoxide, which subsequently undergoes a dismutation catalyzed by the superoxide dismutases SOD1 and SOD2 located in the intermembrane space and mitochondrial matrix, respectively [20,21]. Both rotenone and FCCP have been shown to increase the generation of ROS by two different mechanisms. Rotenone disrupts the transfer of electrons from the iron-sulfur centers in complex I to ubiquinol, resulting in electrons leaking into the mitochondrial matrix and the subsequent building up of ROS. FCCP, on the other hand, depolarizes mitochondria increasing oxygen free radical species, which has been reported in both human endothelial and pulmonary adenocarcinoma cell types [22,23]. ROS, especially at high levels, have been shown to induce the formation of mitochondrial permeability transition pores (mPTP), a key positive regulator in releasing the ROS burst [24] that may degrade adherent junction proteins (AJP). Importantly, although AJPs do not physically impede the paracellular diffusion of macromolecules, they have been shown to indirectly alter the integrity of tight junction proteins [25]. Of these AJPs influenced by ROS generation are β-catenin and E-cadherin located directly under the zonula occludens (ZO-1), an important tight junction protein [26]. The generation of ROS has been linked to the dissociation of β-catenin and E-cadherin from each other as well as from the relocation of both proteins from intercellular junctions to intracellular compartments [27]. Previous reports have shown that in retinal pigment epithelial (RPE) cells exposed to increased levels of an oxidizing agent, there were losses in paracellular attachments; specifically, β-catenin, ZO-1, and E-cadherin were being disrupted [28,29]. Given these findings, a possible explanation of complex I inhibition or mitochondrial uncoupling-mediated disruption of the paracellular barrier function of HREC can be attributed to the damage of AJPs by ROS.

Surprisingly, HREC death does not account for the complex I inhibition-mediated breakdown effect on cell–cell junctions, as rotenone-treated HRECs remained viable even while losing resistance. Aside from ROS, other processes have been reported to be involved in the rotenone-induced disruption of intracellular connections between HRECs. A new study using kidney tubular epithelial cells [30] showed that rotenone stimulates the expression of vascular endothelial growth factor (VEGF), which then activates the *tyrosine* kinase receptor, VEGF receptor-2 (VEGFR2). The ZO-1 [31], specifically its conserved tyrosine residues [32], may be targeted by activated epithelial VEGFR2. We and others have demonstrated that R_b_ measurement changes are correlated with ZO-1 expression changes [15,33]. Therefore, it is suggested that phosphorylation of ZO-1 may be a key in the remodeling of intercellular junctions in HRECs in response to complex I inhibition. As further support, a recent *in vivo* experiment showed reduced and disrupted levels of ZO-1 expression in the epithelial lining of the colon in rotenone-treated mice [34].

Another mechanism that may elucidate the process of rotenone-induced disruption of intracellular connections in HRECs is the mitotic catastrophe coupled with mitochondrial DNA damage and increased activity of autophagy [35]. Induced autophagy has been shown to disrupt cells’ tight junctions in endothelial cells of the blood–brain barrier, another potent barrier system, by downregulating tight junction proteins claudin-5, occludin, and ZO-1 [36]. In support, dysregulated autophagy has been related to the pathophysiology of age-related macular degeneration and diabetic retinopathy [37,38].

Interestingly, we observed that when HRECs were treated with FCCP 1 µM, there was undulating behavior to the total impedance in the middle of the experiment prior to a return to a steady decent (Figure 3A). It has been demonstrated that the endogenous uncoupling protein UCP-2 has a protective role against the accumulation of ROS [39]; therefore, this could explain the brief recovery of HREC barrier function after FCCP treatment. Perhaps there is a partial protective function of the uncoupling proteins against generated ROS that occurs before the cell is overwhelmed and barrier functions dissolve. Additionally, we noted that FCCP weakened the strength of HREC attachment to its substrate, which was evident by a decrease in the α (AUC of normalized value), but no changes were observed in α when HRECs were treated with oligomycin. Generally, surface integrins bound to extracellular matrix (ECM) proteins control cell adhesion to the substrate and hence control α in epithelial cells [40,41]. Recent literature shows the importance of integrin-linked kinase (ILK) in the adhesion process and the role of ATP bound to this pseudokinase. For ILK to retain focal adhesion stabilization, ATP is a necessary binding component [42]. On the other hand, our study shows that the uncoupling of OxPhos, but not ATP synthase inhibition, resulted in less cellular adhesion of HERCs to their substrate, which outlines a new finding and key difference between endothelial and epithelial cells.

To describe the mechanisms related to FCCP-induced changes in C_m_ and therefore changes to the HRECs transcellular barrier, the disruption of ion conductance at the cell membrane is a relevant factor. For example, FCCP can alter the conductance of potassium ions through inward rectifying K+ channels by acidifying the cytoplasm [43,44], resulting in temporary activation followed by inhibition [45]. It is also possible that sodium and hydrogen conductance is altered as FCCP has been shown to increase their currents across plasma membranes in bovine aortic endothelial cells [46]. Another ion channel permeability perturbation mediated by FCCP to consider is that of calcium, as FCCP can release this ion from the mitochondria into the cytoplasm of neuroendocrine cells, where calcium release leads to considerable increases in capacitance [47].

When comparing the effects of the three mitochondrial inhibitors on the various resistance parameters between RPE cells in our previous study [17] to HRECs in the present work, there are key similarities and differences that need to be highlighted. In RPE cells, rotenone disrupted R_b_ in a dose-dependent fashion, while in HRECs, we showed that a single concentration of 1 µM rotenone completely abolished R_b_. In terms of OxPhos uncoupling, FCCP disrupted all three resistance components (R_b_, C_m_, and α) in a dose-dependent manner in RPE cells, while in HRECs, FCCP resulted in a decrease in R_b_ only after 2.5 h and an increase in C_m_ with a minimal effect on α. These comparisons underscore the importance of OxPhos coupling in maintaining the barrier integrity of RPE cells, while in HRECs, complex I functionality is more vital for barrier integrity. These findings may set the stage for designing personalized treatment options for patients with retinal diseases, depending on if the pathology is primarily related to retinal epithelial or endothelial cell dysfunction.

In conclusion, our results reveal varying roles for three main OxPhos components in maintaining HREC functionality in which complex I of ETC is the key factor controlling barrier integrity of HRECs. This differential effect could serve as a robust screening tool for candidate agents that improve the efficacy of complex I to be used clinically in treating endothelial-related retinal disorders.

## Data Availability

Data associated with this manuscript are available upon request from corresponding author.
